# Lawn mowing frequency in suburban areas has no detectable effect on Borrelia spp. vector *Ixodes scapularis* (Acari: Ixodidae)

**DOI:** 10.1371/journal.pone.0214615

**Published:** 2019-04-03

**Authors:** Susannah B. Lerman, Vincent D’Amico

**Affiliations:** 1 Northern Research Station, USDA Forest Service, Amherst, Massachusetts, United States of America; 2 Department of Environmental Conservation, University of Massachusetts, Amherst, Massachusetts, United States of America; 3 Northern Research Station, USDA Forest Service, Newark, Delaware, United States of America; University of Kentucky College of Medicine, UNITED STATES

## Abstract

Forests have become increasingly fragmented throughout the US, with residential development serving as the primary driver of these changes. These altered landscapes have provided suitable conditions for a broad range of wildlife, including blacklegged ticks and their hosts. Lawns dominate residential landscapes, and thus their management has the potential to reduce the likelihood of contact with ticks in residential yards. We tested the hypothesis that lawn mowing frequency influences tick occurrence in 16 suburban yards in Springfield, MA. We conducted 144 tick drags in lawns of various lawn mowing frequencies (mowed every week, every 2-weeks and every 3-weeks) and did not collect any ticks of any species. Promoting frequent mowing (i.e., shorter lawns) and the removal of grass clippings could have minimal impacts on tick microhabitats, but is consequential for beneficial wildlife and other ecosystem services associated with urban biodiversity.

## Introduction

Large tracts of contiguous forests have become increasingly fragmented throughout the US, with residential development serving as the primary driver of these changes [1]. Residential development fractures the landscape, interspersing novel habitats such as yards (e.g., lawns, ornamental and exotic plants, vegetable gardens) amidst forest fragments and forest patches [2]. Although many species have disappeared from residential areas [3], these altered landscapes have provided suitable conditions for a broad range of wildlife, including white-tailed deer (*Odocoileus virginianus*), the preferred host for blacklegged ticks *Ixodes scapularis* [4] and white-footed mice (*Peromyscus leucopus*), the reservoir host for the bacterium *Borrelia burgdorferi* [5,6]. When hosting *B*. *burgdorferi* these ticks can transmit the bacteria to the bitten person, resulting in *Lyme borreliosis* (Lyme disease), a tick-borne infection that is prevalent throughout the northeastern US [7]. In the 10-year period between 2006 and 2015, confirmed cases of Lyme disease in the US reached a mean of 8.1 cases per 100,000 individuals. In Massachusetts, the focal area of our study, over 30,000 confirmed cases were reported during the same period [8]. The urban/suburban environment of Springfield MA, our study area, might appear poor habitat for white-tailed deer, but hunters harvested over 700 deer in the Springfield area management zone in 2017 (MA Division of Fisheries and Wildlife, https://www.mass.gov/service-details/deer-harvest-data).

Identifying opportunities to mitigate contact with ticks in residential landscapes presents an important public health issue. Since lawns dominate the vegetation component of yards [9] altering their management could help reduce contact. Consumer Reports, a non-profit organization that researches and tests products and services provided five recommendations for discouraging ticks from private properties, with two recommendations focusing on lawn mowing: 1) let grass grow to 10.2 cm– 11.4 cm, then cut to 7.6 cm and 2) remove grass clippings [10].

A body of literature exists that anecdotally recounts the dangers of acquiring tick bites and subsequent Lyme disease through exposure to lawns, but the scientific literature has more nuanced results. Ticks found “in” lawns in studies were closely associated with adjacent woodlands [11–13], or represented part of a pooled sample that included woodland edge and other habitats [14]. Meanwhile, other studies that distinguished between different habitat features in residential landscapes have demonstrated a negative relationship between lawn presence and tick abundance, and a positive relationship with woodlands [4,13]. The woodlands, particularly in urban and suburban areas, consist of small forests with mature trees, understory shrubs and leaf litter, with the shrub and litter providing good habitat for ticks [6].

In a study assessing management applications for improving pollinator habitat in lawn-dominated yards, Lerman et al. [15] demonstrated that lawns mowed less frequently, with grass height averaging 12.5 cm supported higher abundances of native bees compared with lawns with grass heights of 11.2 cm. Thus managing for the removal of pest species (e.g., ticks) could have negative impacts for beneficial species (e.g., pollinators). As part of the broader scope of the investigation on the impacts of lawn management behavior on biodiversity and ecosystem function, Lerman et al. [15] surveyed for ticks, recognizing the public concern taller grasses might pose for ticks. If the taller grasses supported higher abundances of bees and ticks, then opportunities to promote pollinator habitat in less frequently mowed lawns might not be widely adopted due to the health risks associated with ticks. Similar to the other studies investigating relationships between lawn mowing frequency and bee diversity [15] and CO_2_ emissions [16], we tested the hypothesis that lawn mowing frequency would influence tick abundance.

## Materials and methods

### Study site

We conducted the study in 16 lawn-dominated yards in Springfield, MA, the third largest city in Massachusetts, USA. The yards were categorized as medium density residential land use and embedded within a suburban matrix. The yards were predominantly comprised of lawns, although some included limited flower borders or hedges, and two yards abutted forest fragments. Yards were not treated with herbicides or watered for the duration of the study. Participating yard parcel size ranged from 0.03 to 0.18 ha. Householders gave permission to conduct the study in their yards.

### Lawn mowing

Lawns were mowed from May through September in 2013 and 2014, using a Toro 19” self-mulching push mower, (mowing height set at 6.35 cm). Grass clippings remained on the lawn. We assigned each yard to a mowing frequency regime: mowed every week, two-weeks or three-weeks to represent the range of typical mowing behaviors (1–2 weeks) to a more extreme (but realistic) frequency (3-weeks; [17]).

### Vegetation measurement

Grass height was measured immediately prior to every mowing event in each yard at three separate locations. We randomly selected and measured the height of three individual swards for a total of nine height measurements per yard per sampling event. These nine replicates were averaged to produce a single grass height per yard per measurement date. We define height as the length of the sward from the soil surface to the sward tip.

### Tick drags

We used BioQuip’s tick drag sailcloth sheet (58 x 114 cm) to document tick abundance in suburban lawns with various grass lengths. Surveys were conducted roughly every three weeks, prior to the mowing event, and coinciding with peak tick presence [18]. Tick drags consisted of a 5-minute drag in three different locations of the yard, coinciding with the grass measurement locations mentioned above. Tick drag sampling is an efficient and accurate method for estimating the abundance of *Ixodes scapularis* in various different landscape settings, including residential properties [19–22]. The drag method has been used with some success for other species occurring in the study area [23].

## Results

Mean grass height prior to mowing for lawns mowed weekly, every two weeks and every three weeks was 11.2 cm, 12.5 cm, and 15.1 cm, respectively. We conducted 144 tick drags over the course of two years (every three weeks between May and September) and did not collect any ticks of any species (Table 1).

**Table 1:** Summary statistics for grass height, and number of ticks detected for each lawn mowing frequency (1-week, 2-weeks, 3-weeks) and for the entire study, regardless of treatment. Tick drags and grass height measurements were conducted at each site, ten times per season in 2013 and 2014 for a total of 144 tick drags and measurements.

	Mowing frequency	Grass height (cm)	Ticks detected (#)
Mean	1 wk	11.20	0
	2 wks	12.52	0
	3 wks	15.06	0
	Study	12.91	0
Minimum	1 wk	6.70	0
	2 wks	7.80	0
	3 wks	9.40	0
	Study	6.70	0
Maximum	1 wk	18.20	0
	2 wks	23.40	0
	3 wks	26.00	0
	Study	26.00	0
Median	1 wk	11.05	0
	2 wks	12.40	0
	3 wks	13.95	0
	Study	12.30	0
Standard error	1 wk	0.42	0
	2 wks	0.59	0
	3 wks	0.70	0
	Study	0.36	0

## Discussion

Our results support previous findings of the lack of ticks in the lawn zone of residential landscapes. A study conducted in Westchester County, NY investigated four distinct zones of residential properties including wood lots, unmaintained edges (the ecotone), ornamental vegetation and lawns, and their propensity to support blacklegged ticks. Less than 2% of the ticks were collected from lawns with the majority collected from the wood lots and ecotone [24]. Duffy et al. [13] also found that for yards in Suffolk County, NY, nymphs were primarily encountered at the ecotone with few encounters on lawns. Blacklegged ticks are highly sensitive to low humidity and dehydration, and rely on habitat which provides opportunities to rehydrate [25]. Together, these results acknowledge the presence of ticks in residential landscapes—but context matters [4]. Both property size and the surrounding matrix have implications for tick presence. For example, larger properties (e.g., > 0.5 ha) are more likely to have wood lots, and hence, more opportunities to encounter ticks [24]. A study of coastal Maine microhabitats showed grasses to be the poorest quality habitat for ticks even in an unmanaged setting [26]. These and other studies suggest that lawns, particularly those with full exposure to sunlight, provide poor habitat for blacklegged ticks.

Tick-borne diseases pose a serious public health risk [27]. The blacklegged tick is now recognized as a vector of three species of Borrelia, a different bacterium causing anaplasmosis, a parasite causing babesiosis, and the Powassan virus [24]. The loss of urban biodiversity and concomitant invasion by nonnative plants also exacerbates the transmission of some tick-borne diseases due to the dilution effect (i.e., the loss of additional vertebrate hosts [28,29]) although this effect varies with landscape scale [6,30]. Further, studies have shown many nonnative plants, particularly understory shrubs, to be especially good tick habitat. Yard management strategies aimed at reducing contact with ticks should consider removing nonnative plants to provide an opportunity for individual households to combat some of the ecosystem disservices associated with forest fragmentation [18]. In addition, identifying where and whether the risk is occurring can help provide support for ensuring individual efforts lead to desired results of fewer interactions with ticks in lawns.

Providing solutions for reducing contact with ticks, such as promoting frequent lawn mowing, is an apparently simple practice with the potential to be widely adopted. However, we suggest that recommendations be supported by research [31], acknowledge the limitations for protecting against ticks, and enumerate the trade-offs associated with frequent mowing. In our study system, taller grasses did not result in more ticks but did support higher abundances and diversity of native bees [15]. Thus, promoting shorter grasses and the removal of grass clippings could have minimal impacts on tick microhabitats but would be consequential for beneficial wildlife such as pollinators, and other ecosystem services associated with urban biodiversity [32].
